# Hemodynamic and metabolic alterations associated with septic acute kidney injury in experimental sepsis

**DOI:** 10.1186/2197-425X-3-S1-A469

**Published:** 2015-10-01

**Authors:** EH Post, F Su, K Hosokawa, FS Taccone, A Herpain, J Creteur, J -L Vincent, D De Backer

**Affiliations:** Erasme University Hospital, Intensive Care Unit, Brussels, Belgium

## Introduction

The role of renal perfusion in the development of septic acute kidney injury (AKI) remains elusive. When septic AKI develops in the presence of hypotension, renal dysfunction is considered to be caused by reduced renal blood flow and tissue hypoxia. However, an integrated view of the effects of sepsis on renal blood flow, oxygenation and local metabolism is currently lacking.

## Objectives

To assess renal perfusion, kidney cortex metabolism and tissue oxygen tension in an ovine model of septic shock.

## Methods

12 animals were randomized to sepsis (n = 8) or sham procedure (n = 4). A pre-calibrated flow probe was positioned around the renal artery to measure renal blood flow (RBF) and a catheter was inserted into the renal vein to measure renal vein oxygen content and calculate renal oxygen consumption, corrected for body surface area (renal VO_2_I). A tissue oxygen-tension electrode and a microdialysis probe were inserted into the kidney cortex to measure interstitial oxygen tension (tPO_2_) and glucose, lactate and pyruvate levels, respectively.

Sepsis was induced by injection of 1.5 g/kg autologous feces into the abdominal cavity (T0h). Sham animals underwent similar surgery but no feces were injected. Treatment consisted of fluid-administration (Ringer's lactate and HES 130/4.2 in a 1:1 ratio) to keep pulmonary artery occluded wedge pressure at baseline levels.

The animals were observed for 18 hours and data were analyzed for main effect of time and interaction between group and time using linear mixed models. In case of significance, pairwise comparisons were carried out using Student's t-test. A p-value of less than 0.05 was considered statistically significant.

## Results

The septic group developed renal dysfunction at T12h, as evidenced by the occurrence of oliguria and a reduced creatinine clearance (table 1), concomitantly to the development of hypotension (MAP 47 ± 15 mmHg in control vs. 81 ± 4 mmHg in sham animals; p < 0.05). Low RBF and reduced renal VO_2_I were also observed after 12 hours. These findings were associated with increased cortical lactate and pyruvate levels and an elevated lactate pyruvate ratio (LPR) at T18h (table 2). In contrast, renal cortex tPO_2_ remained unchanged in both groups during the observation period.

**Table 1:** Renal hemodynamics and function.

		T0h	T6h	T12h	T18h
RBF, mL/min	Sepsis	165 ± 32	138 ± 84	71 ± 38 #,*	45 ± 23 #,*
	Sham	175 ± 47	185 ± 47	188 ± 51	198 ± 61
Renal VO_2_I, mL/min/m^2^	Sepsis	2.1 ± 0.6	1.9 ± 0.7	1.2 ± 0.8 *	0.9 ± 0.5 #,*
	Sham	1.6 ± 0.6	1.6 ± 0.5	1.7 ± 0.4	1.9 ± 0.5
Creatinine Clearance, mL/min	Sepsis	77 ± 25	66 ± 37	12 ± 0.6 #,*	3 ± 2 #,*
	Sham	55 ± 12	53 ± 24	51 ± 15	52 ± 27
UO, mL/kg/hour	Sepsis	2.2 ± 1.1	1.8 ± 0.8	0.3 ± 0.2 #,*	0.1 ± 0.1 #,*
	Sham	1.5 ± 0.8	1.6 ± 0.9	1.6 ± 0.8	1.6 ± 0.9

**Table 2:** Renal cortex metabolism.

		T0h	T6h	T12h	T18h
Glucose, mg/dL	Sepsis	21 ± 7	20 ± 12	20 ± 13	16 ± 17
	Sham	28 ± 14	18 ± 5	19 ± 5	22 ± 5
Lactate, mmol/L	Sepsis	0.5 ± 0.3	0.7 ± 0.3	1.4 ± 0.7 #,*	4.1 ± 1.6 #,*
	Sham	0.4 ± 0.1	0.7 ± 0.3	0.6 ± 0.3	0.4 ± 0.2
Pyruvate, μmol/L	Sepsis	23 ± 12	44 ± 12 *	69 ± 33 #,*	59 ± 48
	Sham	29 ± 9	41 ± 18	38 ± 18	28 ± 14
Tissue PO_2_, mmHg	Sepsis	47.0 ± 7.3	46.8 ± 15.2	42.9 ± 10.8	43.9 ± 14.6
	Sham	41.7 ± 3.9	52.5 ± 6.3	50.9 ± 5.3	51.9 ± 5.8

## Conclusions

In this model of experimental sepsis, severe AKI was associated with marked reduction in renal perfusion and metabolism although cortical PO_2_ was preserved.

